# Efficacy of three-dimensionally integrated exercise for scoliosis in patients with adolescent idiopathic scoliosis: study protocol for a randomized controlled trial

**DOI:** 10.1186/s13063-018-2834-x

**Published:** 2018-09-10

**Authors:** Juping Liang, Xuan Zhou, Nan Chen, Xin Li, Hong Yu, Yuqi Yang, Yuanyuan Song, Qing Du

**Affiliations:** 1 Department of Rehabilitation Medicine, Xinhua Hospital affiliated to Shanghai Jiao Tong University School of Medicine, Shanghai, 200092 China; 2 School of Nursing and Health Management, Shanghai University of Medicine & Health Sciences, Shanghai, 201318 China; 3 Department of Rehabilitation Medicine, Xinhua Hospital affiliated to Shanghai Jiao Tong University School of Medicine, Chongming Branch, Shanghai, 202150 China

**Keywords:** Adolescent idiopathic scoliosis, Three-dimensionally integrated exercise, Cobb angle, ATR, Sagittal profile, Quality of life

## Abstract

**Background:**

Adolescent idiopathic scoliosis (AIS) is one of the most prevalent spinal deformities that may progress sharply during growth. The aim of this study will be to evaluate the efficacy of three-dimensionally integrated exercise on the Cobb angle, angle of trunk rotation, sagittal profile, and quality of life in patients with AIS.

**Methods/design:**

The study is designed as a randomized controlled trial. Participants include 42 patients with AIS aged 10–16 years. Randomly assigned patients will follow a 6-month treatment, either in a control group with standard care of observation following the Scoliosis Research Society criteria or in an experimental group with three-dimensionally integrated exercise for scoliosis. Blinded assessments at baseline and immediately after intervention will include the change of Cobb angle, angle of trunk rotation, sagittal index, and quality of life.

**Discussion:**

If we find that the intervention is effective in improving Cobb angle, angle of trunk rotation, sagittal profile, and quality of life in patients with AIS, this trial will have a positive impact and warrant a change in clinical practice.

**Trial registration:**

ClinicalTrials.gov, NCT03427970. Registered on February 9, 2018, and revised on July 24, 2018.

**Electronic supplementary material:**

The online version of this article (10.1186/s13063-018-2834-x) contains supplementary material, which is available to authorized users.

## Background

Scoliosis is a three-dimensional deformity with lateral curvature, sagittal hypokyphosis, and axial rotation of the spine. Adolescent idiopathic scoliosis (AIS) with a Cobb angle greater than 10 degrees occurs in the general population with a frequency ranging from 2% to 5% during growth, and the origin of the disease is unknown [1–4]. It has been highlighted that impairment of esthetic appearance of a patient’s body is significantly related to the patient’s negative image and self-respect, and the disease may cause reduced bone strength and balance disorder, among other effects [5–7]. If uncontrolled, scoliosis can progress in a short time, and moreover, severe trunk deformity, cardiopulmonary impairment, and low quality of life may occur [1, 8]. There is still no intervention to cure scoliosis [1, 8].

According to the severity of the curve, the major treatment approaches for patients with AIS include exercises, bracing, and surgery to correct, prevent, or stop the progression of the deformity [1, 8, 9]. In North America, the Scoliosis Research Society (SRS) has published the standard of care for AIS: Patients with curvature between 10 degrees and 25 degrees who are still growing should be kept under observation [9]. In Europe, the International Scientific Society on Scoliosis Orthopaedic and Rehabilitation Treatment has recommended that physiotherapeutic scoliosis-specific exercises should be the first step in treating idiopathic scoliosis to prevent/limit the progression of the deformity [1, 8]. Three-dimensionally integrated exercise for scoliosis is based on the theory of physiotherapeutic scoliosis-specific exercises and consists of patient education, three-dimensional self-correction, stabilization of the corrected posture, and training activity of daily living, combined with neuromotor control, proprioceptive training, balance training, and so forth, tailoring the exercise approach individually for each patient [1, 8, 10]. The specific exercises could accelerate the recovery of physiology, psychology, and functional ability for patients with AIS as compared with other approaches, but these outcomes may take a long time to achieve [11–19].

The Cobb method is the gold standard for measuring the scoliotic curve clinically [20], and the trunk rotation and sagittal profile are also used to assess the trunk deformity and the physiological curvature for patients with spinal diseases [21–23]. Normally, different interventions will achieve varied satisfaction levels related to treatment and quality of life for patients [24]. Limited evidence from 3-month studies of three-dimensionally integrated exercise for scoliosis have confirmed its positive value in preventing the curve progression, maintaining the corrected posture, and improving patients’ quality of life [25, 26]. However, better-quality research needs to be conducted before the use of three-dimensionally integrated exercise for scoliosis can be recommended in clinical practice. Therefore, the objective of this trial is to determine the effect of intervention on the Cobb angle, angle of trunk rotation (ATR), sagittal profile, and quality of life compared with standard care for patients with AIS.

## Methods/design

### Aims and objectives

The aim of this study is to assess the effect of the intervention on Cobb angle, ATR, sagittal profile, and quality of life in patients with AIS.

### Study design

**Fig. 1:**
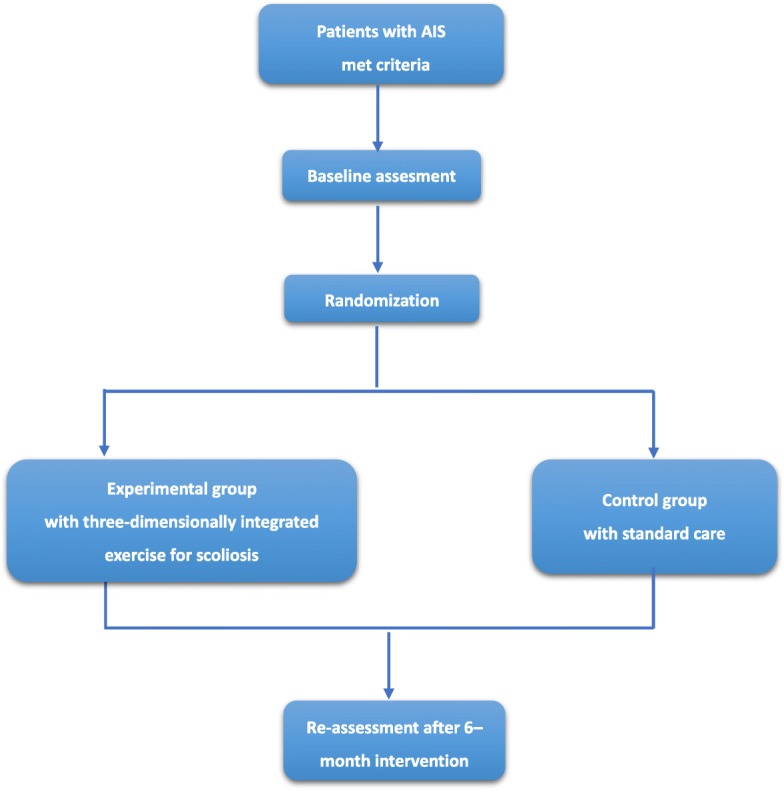
Trial profile

The present study is a randomized controlled trial (RCT) comparing a 6-month three-dimensionally integrated exercise for scoliosis intervention with a standard of care of observation alone (control group). The protocol conforms to Consolidated Standards of Reporting Trials (CONSORT) guidelines for nonpharmacological studies [27, 28] and the World Health Organization Trial Registration Data Set Version 1.2.1 guidelines for clinical trials protocols (See in Table 1 and Additional file 1). The study is being conducted at the Department of Rehabilitation Medicine, Xinhua Hospital affiliated to Shanghai Jiao Tong University School of Medicine, China. A trained research assistant who is blinded to the randomization design will invite eligible patients to attend the specific scoliosis clinic to participate in the study. The physician in the clinic will explain the whole protocol in the quiet clinic room and obtain written informed consent from each participant and legal representative prior to inclusion. The trial profile is displayed in Fig. 1.

**Table 1:** World Health Organization Trial Registration Data Set (Version 1.2.1)

Item number	Item	Description	Addressed on page number
1	Primary registry and trial identifying number	Name of primary registry and the unique identifier assigned by the primary registry	3
2	Date of registration in primary registry	Date when the trial was officially registered in the primary registry	3
3	Secondary identifying numbers	Other identifiers, if any Universal Trial Number Identifiers assigned by the sponsor Other trial registration numbers issued by other registries Identifiers issued by funding bodies, collaborative research groups, regulatory authorities, ethics committees/institutional review boards, etc.	Not applicable
4	Sources of monetary or material support	Major sources of monetary or material support for the trial (for example, funding agency, foundation, company, and institution)	17
5	Primary sponsor	Person, organization, group, or other legal entity that takes responsibility for initiating and managing a study	17
6	Secondary sponsor(s)	Additional persons, organizations, or other legal persons, if any, who have agreed with the primary sponsor to take on responsibilities of sponsorship	Not applicable
7	Contact for public queries	E-mail address, telephone number, and postal address of the contact who will respond to general queries, including information about current recruitment status	2
8	Contact for scientific queries	Name and title, e-mail address, telephone number, postal address, and affiliation of the principal investigator and e-mail address, telephone number, postal address, and affiliation of the contact for scientific queries about the trial (if applicable)	2
9	Public title	Title intended for the lay public in easily understood language	1
10	Scientific title	Scientific title of the study as it appears in the protocol submitted for funding and ethical review; include trial acronym, if available	Not applicable
11	Countries of recruitment	Countries of recruitment Countries from which participants will be recruited	5
12	Health condition(s) or problem(s) studied	Primary health condition(s) or problem(s) studied (for example, depression, breast cancer, or medication error)	4
13	Intervention(s)	For each group of the trial, record a brief intervention name plus an intervention description name. For drugs, use the generic name; for other types of interventions, provide a brief descriptive name of the intervention. This name must be sufficiently detailed for it to be possible to distinguish between the groups of a study; for example, interventions involving drugs may include dosage form, dosage, frequency, and duration	5–10
14	Key inclusion and exclusion criteria	Inclusion and exclusion criteria for participant selection, including age and sex	7–8
15	Study type	Method of allocation (randomized/nonrandomized) and blinding/masking (identify who is blinded) Assignment (for example, single-group, parallel, crossover, or factorial) and purpose Phase (if applicable) For randomized trials, method of sequence generation and allocation concealment	5
16	Date of first enrollment	Anticipated or actual date of enrollment of the first participant	16
17	Target sample size	Total number of participants to enroll	13
18	Recruitment status	Pending: Participants are not yet being recruited or enrolled at any site Recruiting: Participants are currently being recruited and enrolled Suspended: Temporary halt in recruitment and enrollment Complete: Participants are no longer being recruited or enrolled Other	16
19	Primary outcome(s)	The primary outcome should be the outcome used in sample size calculations or the main outcome used to determine the effects of the intervention For each primary outcome, provide the following: Name of the outcome (do not use abbreviations) Metric or method of measurement used (be as specific as possible) Time point of primary interest	10
20	Key secondary outcome(s)	As for primary outcomes, for each secondary outcome, provide the following: Name of the outcome (do not use abbreviations) Metric or method of measurement used (be as specific as possible) Time point of interest	10–12

### Participants

#### Inclusion criteria

1. A diagnosis of AIS [8]
2. Aged 10 to 16 years
3. A Cobb angle of 10–20 degrees
4. A Risser sign of 0–3
5. No other treatment that might affect scoliosis

#### Exclusion criteria

1. Nonidiopathic scoliosis, which is caused by neuromuscular disorder, vertebral malformation, trauma, tumor, or other diseases
2. Accompanying psychiatric problems or neuromuscular or rheumatic diseases
3. Previous surgical history involving the spine or lower extremities
4. Previous exercise or brace treatment history
5. Having contraindications to exercise

### Randomization and masking

After an initial assessment examination confirming eligibility and collection of baseline data, participants will be allocated by a physician using computer-generated block randomization in a ratio of 1:1 to the experimental group or control group. The allocations will be concealed in sequentially numbered, opaque, sealed envelopes with a signature across the sealing point.

**Fig. 2:**
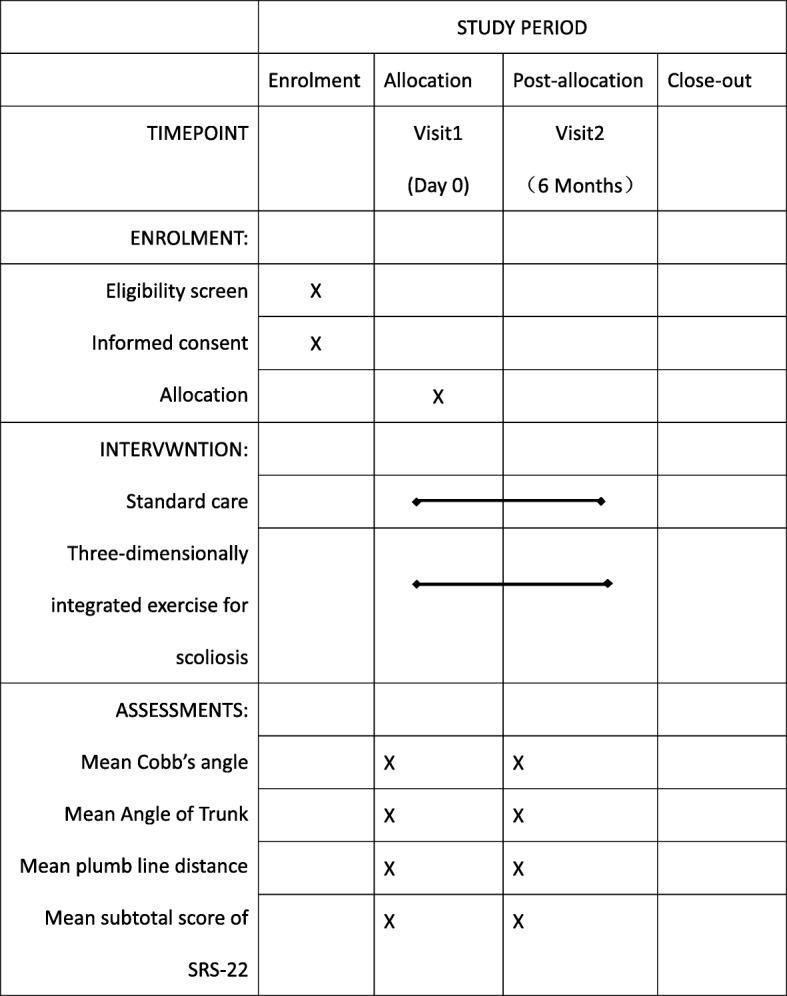
Standard Protocol Items: Recommendations for Interventional Trials (SPIRIT) figure

Because of the nature of the intervention, physical therapists and patients cannot be blinded when offering or receiving the treatment. However, patients will be asked not to reveal their group allocation to ensure blinding of the evaluator. To minimize bias, strict eligibility will be maintained in the whole trial, and there are separate rooms for assessment and treatment, so that each group will hardly know much about each other. A trained evaluator blinded to group allocation will complete all patient assessments at baseline and immediately after the 6-month intervention. Both groups will continue with their routine activities, but they will not be able to attend any other formal exercise program. The statistician will not be aware of the data coding. Figure 2 illustrates the timing of all trial processes (Additional file 2).

### Intervention

#### Experimental intervention program

**Fig. 3:**
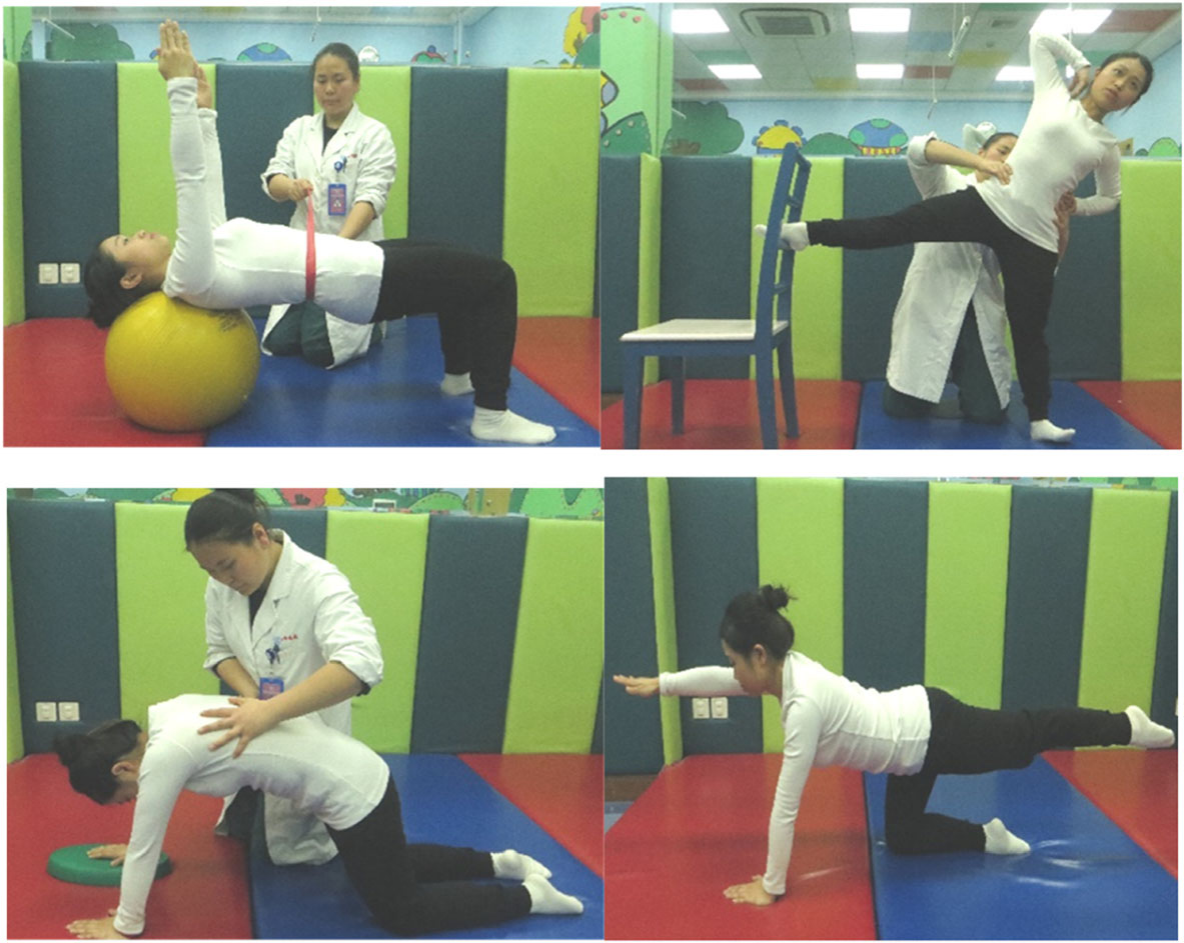
Examples of three-dimensionally integrated exercise for scoliosis

The experimental group will receive a modified physiotherapeutic scoliosis-specific exercise intervention: the three-dimensionally integrated exercise for scoliosis program, which includes autocorrection in three dimensions, postural correction, breathing training, resistance training, muscle fascia releasing, functional activities, balance training, core stability training, proprioceptive input exercises, and patient education. Autocorrection exercise in three dimensions will be combined with specific breathing mode, and isometric contraction training to correct abnormal spinal physiological curvatures in the sagittal plane, accompanied by a wedge pad to modify humpback, waist asymmetry, and pelvic rotation in the horizontal plane. In breathing pattern training, the physical therapist will stimulate the concave areas of the thorax for patients during deep inspiration and will transfer stimulation to the convex side during deep expiration. While the patient is in coronal plane with longitudinal axial stretching, pelvic adjustments will be made to reduce the lateral curvature. During this period, the physical therapist will teach the patient how to combine these exercises with activities of daily living. All the patients in the experimental arm will receive treatment of one or two sessions weekly in the hospital under the supervision of a physiotherapist and one session per day at home under parental supervision. Some examples are shown in Fig. 3.

The physical therapist has 3 years of scoliosis therapy experience and will provide 95% of the therapy sessions. Another certified physical therapist will fill in as needed.

### Control group program

Control subjects will receive the standard of care according to the SRS criteria: observation for patients with curves between 10 degrees and 25 degrees [9] and attendance at the study assessments. The control group will only attend study assessments and not the therapy sessions.

### Compliance supervision

Compliance will be monitored using a home training diary to record the time and frequency of every-day home training, verified daily by a parent and weekly by a therapist. Meanwhile, all the patients will be added to Micro Message Platform app created by the research assistant. The assistant will divide the patients into two groups following their allocation and check the daily training at irregular intervals, answer patients’ questions online, share health knowledge with the patients, or remind the patients to attend clinic visits after the 6-month intervention. To maximize compliance, the physical therapist will provide home equipment, provide the patients with access to facilities, change the exercises to improve compliance with exercises to avoid patients’ getting bored with always performing the same exercises, and encourage parents to be fully engaged to ensure the exercises are regularly performed at home. Attendance will be calculated as a percentage of the prescribed visits attended, and compliance will be calculated as a percentage of the prescribed home exercise dose completed over the course of the 6 months of treatment. When compliance drops below 70%, the physical therapist will try to resolve the issues cooperatively with patients and parents [29, 30].

### Withdrawal criteria

The patients or their families will be allowed to withdraw from the study if they make such a request.

### Suspension criteria

In the interim analysis, clinical significance is defined as a 5-degree Cobb angle change, which is the reliability of radiographic examination and the international “gold standard” for minimally significant clinical change [31]. Decreasing or remaining in Cobb angle of 5 degrees means the treatment is successful, whereas increasing the Cobb angle to greater than 5 degrees is defined as failure of treatment [32]. There are no standard stopping criteria for specific exercise therapy outcome. Previous studies showed that there is a 40% failure rate for standard care (observation) [18]. In light of the clinical practice as well as the previous studies, the stopping criteria used in this protocol are as follows:

1. If more than 40% of patients fail in either the experimental group with three-dimensionally integrated exercise for scoliosis or the control group, the trial will be ended, suggesting a change of treatment.
2. During the whole study period, if the patient has significantly abnormal progression in clinical examination and the Cobb angle has reached ≥ 25 degrees, the patient (in either the experimental group or the control group) will be suspended from the study.

### Assessment

The following demographic data and patient characteristics of the experimental group will be collected: age, gender, height, weight, body mass index, Risser sign, time since menarche for female patients, curve pattern, apex levels, and exercise compliance. Skeletal immaturity based on the Risser stages will be recorded for each patient at the first assessment. Curvatures will be classified according to the Ponseti classification system, which distinguishes four major types of scoliosis: thoracic, lumbar, thoracolumbar, and S-shaped [8, 33]. Development of the ossification of the iliac crest is used to assess the remaining spinal growth, and the stage of ossification of the iliac apophysis can be seen on a radiograph. The Risser sign is an assessment of skeletal maturity and spinal growth. It is based on the ossification of the iliac apophysis, which is evaluated with a four-grade scale from 0 (no ossification) to 5 (fused ossified apophysis) [34]. The outcome measures described in the subsections that follow will be performed before and after 6 months of intervention in both groups.

### Primary outcome measurement

#### Change of Cobb angle

**Fig. 4:**
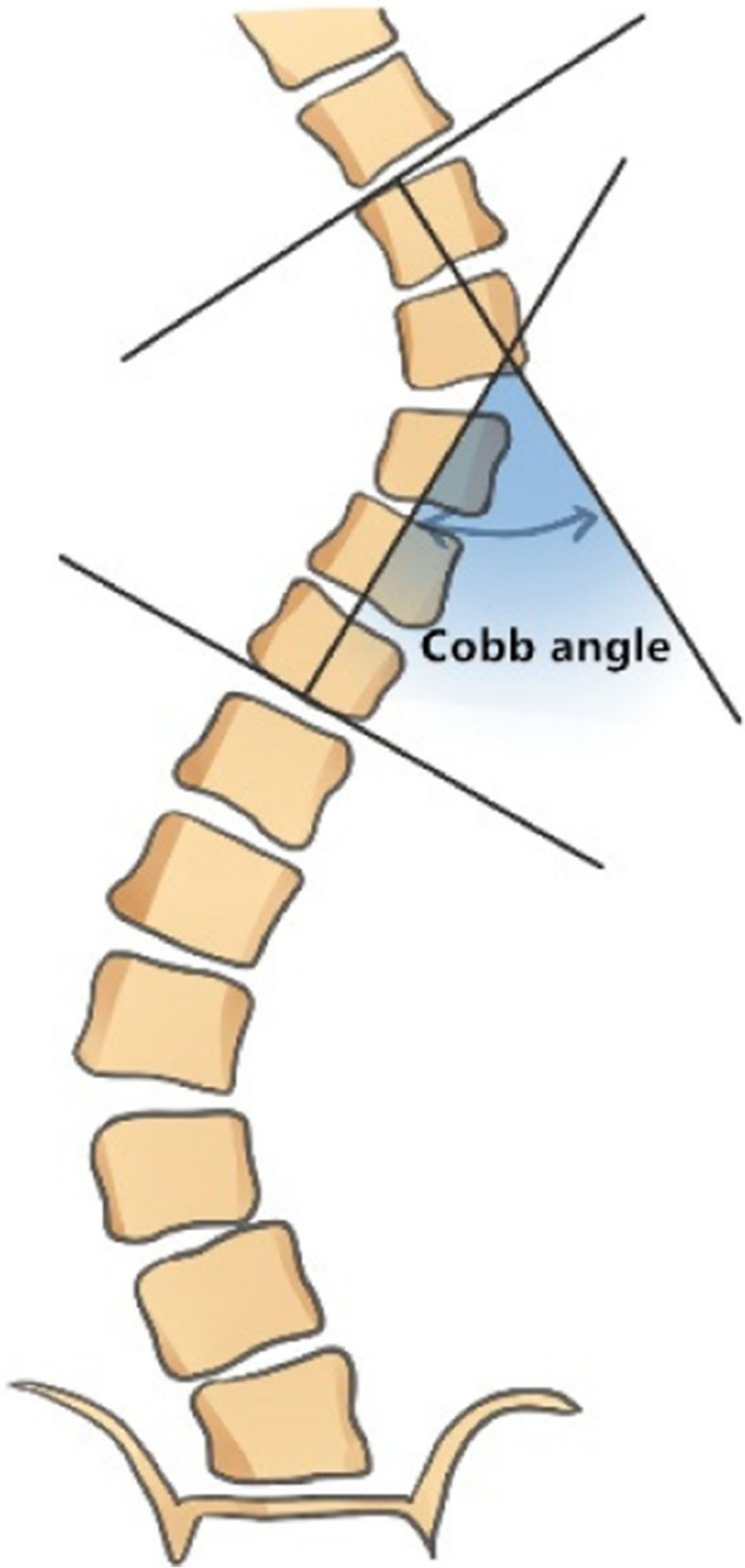
The Cobb method of measurement

It is recommended that curve magnitude be measured using the Cobb method on a standing frontal radiograph [20]. The Cobb angle of a scoliotic curve is defined as the angle formed by the intersection of two lines, one parallel to the endplate of the superior end vertebra and the other parallel to the endplate of the inferior end vertebra (Fig. 4). If the patient has double curves, the major curve will be calculated for analysis.

### Secondary outcome measurements

#### Angle of trunk rotation

**Fig. 5:**
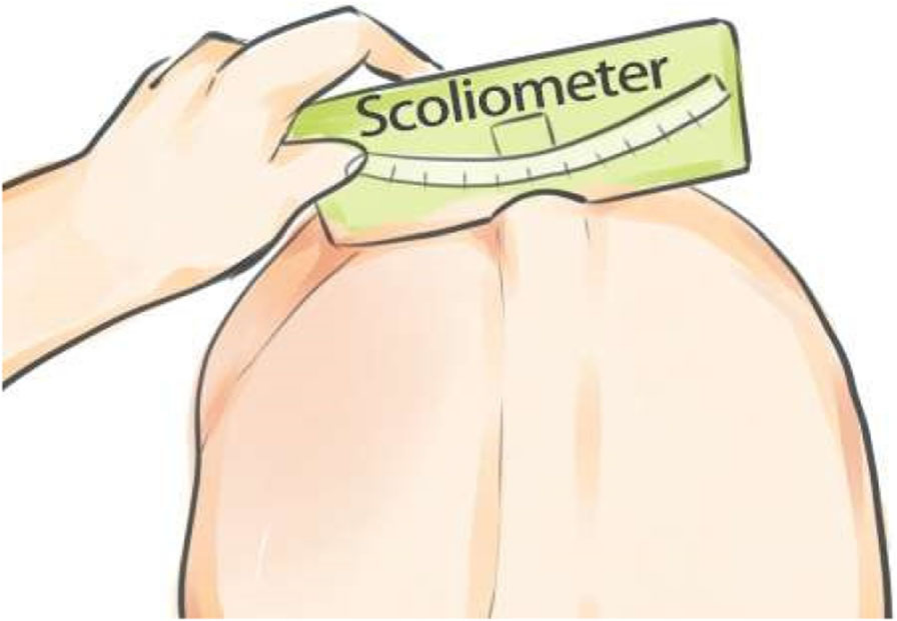
Angle of trunk rotation

The ATR will be measured with a Scoliometer™ [21, 22]. The Scoliometer™ measures the hump (ATR) appearing as a consequence of the Adam’s test. It is an evaluation tool that has proven highly useful [1, 8]. The patients will be asked to bend their trunk forward until it is parallel to the ground, keeping the palms of their hands together with their arms hanging down and perpendicular to their trunk. In this position (Fig. 5), the ATR values are obtained by positioning the center of the Scoliometer™ over the spinous process and perpendicular to the spine [35].

#### Sagittal index

**Fig. 6:**
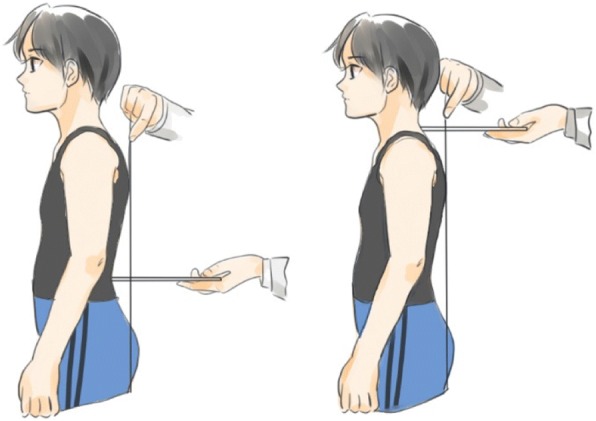
Measurement of sagittal distance

The sagittal profile of the spine is frequently modified in patients with scoliosis and is extremely relevant in the management of this disease; thus, a sagittal measurement is recommended to evaluate kyphosis and lordosis [36]. The plumb line is an easy, quick, and reliable tool to assess the sagittal profile in clinical evaluation and clinical follow-up, as well as for the evaluation of the effectiveness of physiotherapy-specific exercises [23]. Measurement of the plumb line distance is measured from the apical spinous process of the occiput, the most prominent lordosis point on the neck, from C7, T5-T6, T12, L3, and S1 to the plumb line (Fig. 6). The sagittal index consists of the sum of the plumb line distances from C7 and L3; a value < 60 mm is considered to be a flat back; 60–90 mm is in the normal range, whereas a value > 90 mm is considered to indicate kyphosis [37, 38].

#### SRS-22 dimension scores

The 22-item Scoliosis Research Society Outcomes Questionnaire (SRS-22) is especially designed for patients with idiopathic scoliosis [24]. The questionnaire consists of 22 items with 5 dimensions: function/activity, pain, self-image, mental health, and satisfaction with treatment. Each domain has five items each, except the satisfaction with treatment domain, which has only two items. The two satisfaction items are not included in the final analysis. Each item is scored from 1 (worst) to 5 (best). Results are presented as the mean of each scale (sum of 5 questions/5) and the mean subtotal score (sum of 20 questions/20) [39, 40]. In this study, we will use the validated Chinese version of SRS-22 [41].

### Adverse events

The physical therapist will record any adverse event occurring in this study.

### Sample measurement

G*Power 3.1.9.2 was used to perform the power calculations. The change of Cobb angle was the primary outcome measurement. The results of our previous study showed that the change of Cobb angle in the experimental group was − 1.55 ± 4.39 degrees, whereas in the control group, it was 2.90 ± 2.60 degrees [25]. The α will be 0.05, and the β will be 0.05. Therefore, we should recruit 19 patients to each group. Considering 10% potential attrition, 21 patients per group will be recruited.

### Data collection, data management, and statistical analysis plan

The primary outcome is the change of Cobb angle, and the secondary outcome measures will include the ATR, sagittal index, and quality of life. Descriptive statistics will be calculated for baseline demographics, physical examination, radiographs, and questionnaire data, and results will be presented as the mean ± SD, whereas categorical variables will be expressed as percentages. The chi-squared test will be used to compare the categorical/dichotomous variables. Separate analyses will be conducted for each outcome.

Data will be entered using EpiData software designed for this study. All data will be collected, typed, and analyzed by a masked statistician who will not have access to study assignment. The main investigators will check the data every 2 weeks to ensure the quality.

The primary endpoint is the pre- and posttreatment difference in the Cobb angle. We have calculated that a sample size of 21 patients per group would be capable of detecting a between-group difference of 5 degrees in the primary endpoint with a type I error of 5% and a power of 95% [20]. Because the interval of Cobb angles at entry is 10–20 degrees, and assuming a uniform distribution of Cobb angles in this range (the most unfavorable scenario), an SD of 5 degrees is estimated [8].

Baseline comparability will be assessed using Student’s *t* test for independent samples. To screen for variables associated with each of the outcome variables, univariate linear mixed models for each relevant covariable (age, gender, body mass index, Risser sign, time since menarche, apex levels, curve pattern, and compliance may have associations with risk of progression) will be conducted.

Multiple linear mixed models for each time point will be tested, including covariables found significant at *p* ≤ 0.20 in the univariate analysis, and after screening for multicollinearity [42].

For the ordinal outcome (SRS-22, which is based on only one item with five levels), generalized linear mixed effects model analysis will be used.

Furthermore, because subjects with younger than 13 years of age are characterized by a higher risk of progression [43], the patients will be divided into two subgroups (age < 13 years and age ≥ 13 years), and a linear mixed model analysis for the primary outcome will be performed on each subgroup.

To assess differences in group changes from baseline to 6 months while adjusting for covariables, weighted estimating equations and a sensitivity analysis will be conducted as the primary approach to deal with the missing data, especially for the primary outcome of Cobb angle [44]. All the analysis will be carried out using IBM SPSS Statistics for version 20.0 software (IBM, Armonk, NY, USA).

### Interim analysis

An interim analysis will be performed on the primary endpoint of Cobb angle when enrolling 20 patients, including control group and experimental group, by a single statistician who is blinded to allocation and reports the results to the main investigators. The main investigators will discuss the results of the interim analysis with the monitoring board. In this study, the change of Cobb angle is the only primary outcome measurement; the secondary outcome measurements are the supporting elements for the whole outcome.

Our primary hypothesis is that the experimental intervention of three-dimensionally integrated exercise for scoliosis will optimize improvement in outcome effects (reduction in Cobb angle and improvement in multifidus activation). Effect sizes will be estimated using Cohen’s *d*, which corresponds to the mean difference between the groups in the change observed from baseline to 6 months (three-dimensionally integrated exercise for scoliosis standard of care), divided by the pooled SD at baseline (Cohen’s *d* ≥ 0.8 = large, 0.5–0.8 = moderate, 0.2–0.5 = small [45]). According to the previous study, the perceived mean global rating of change in the specific exercise group was 3.8 ± 2.2, corresponding to moderate improvement, and 0.3 ± 1.7 in the standard care group, corresponding to a small amount of deterioration [46]. In this study, an effect size change of 0.3 would be considered a significant finding to stop the study when performing the interim analysis [47]. The reasons for withdrawal from trial by arm will be calculated by the physical therapist.

### Harms

If there is a reasonable suspected causal relationship with the intervention, the adverse events will be reported to the ethics committee to guarantee the safety of the patients. We do not expect that there will be any risks for either group (patients with or without intervention).

### Data monitoring and auditing

A monitoring board, including independent assessors (not involved in the study) from the Shanghai Jiao Tong University School of Medicine, will review all data and can conduct an audit of the trial at any time.

### Confidentiality

Only the main investigators will be allowed access to the EpiData software data with passwords. All patients with AIS will be identified by sex, birth date, and evaluation date and will be assigned a trial number during and after the trial in accordance with personal data protection laws.

### Access to data

The main investigators will have the right to access the final and complete trial dataset, and there is no contractual agreement to limit such access to all the investigators.

### Ancillary and posttrial care

After completing the trial, we will continue to evaluate and treat the patients in the future according to their wishes.

### Dissemination policy

The final results of the trial are planned to be published in a scientific journal and presented at medical conferences. We will follow the CONSORT statement guidelines updated in 2010 (http://www.consort-statement.org) and their extension to nonpharmacological interventions or pragmatic trials.

## Discussion

This study protocol describes an RCT that is designed to evaluate the efficacy of three-dimensionally integrated exercise on scoliosis measured as Cobb angle, the ATR in degrees, sagittal profile, and quality of life in patients with mild AIS.

In this study, the patients and intervention team are not blinded, because the intervention team needs to carry out the detailed treatment plan with the patients, and the patients in each group know their treatment plan. The assessor, enrollment assistant, and statisticians are blinded with respect to the patients’ treatment allocation. Meanwhile, there are separate rooms for assessment and treatment to avoid having other patients know about the treatment. The control group only needs to perform the assessment at the baseline and after the 6-month intervention, so they will have less time to communicate with the other patients. Once the patient asks to change the treatment plan, they will be eliminated from the study immediately.

Preventing the curve progression is the most important reason for treatment. There is still no intervention to cure AIS [1, 8]. The results of this RCT will provide valuable information for the family, patient, clinicians, and clinical decision makers, as well as to administrative stakeholders, and they may play an important role in providing healthcare services.

### Strengths and limitations

#### Strengths

1. The three-dimensionally integrated exercise treatment for scoliosis is an individual approach for patients with mild deformity. A matured method of assessment and treatment using three-dimensionally integrated exercise for scoliosis has been set up and supplemented over several years, which has shown a very good outcome.
2. There is a lack of high-quality evidence to support this valuable treatment of three-dimensionally integrated exercise for scoliosis. This study will be the first high-quality trial to confirm its effectiveness.
3. The highlight of three-dimensionally integrated exercise for scoliosis is that it will not be limited to the medical center, but will also affect the patient’s daily life at home or at school.
4. This study focuses on not only the Cobb angle but also the improvement in trunk rotation, sagittal profile, and quality of life.

#### Limitations

1. The total treatment period of 6 months is short.
2. Although the training diary will help with recording the home training situation, this study cannot ensure the quality of exercises done at home.

### Study duration/trial status

This trial is recruiting now. Recruitment of study patients commenced on 26 February 2018.

## Additional files

Additional file 1: World Health Organization Trial Registration Data Set. (PDF 152kb)

Additional file 2: Standard Protocol Items: Recommendations for Interventional Trials (SPIRIT) checklist. Complete SPIRIT checklist. (DOC 271 kb)

## Abbreviations

AIS: Adolescent idiopathic scoliosis
ATR: Angle of trunk rotation
RCT: Randomized controlled trial
SRS: Scoliosis Research Society
SRS-22: 22-item Scoliosis Research Society Outcomes Questionnaire
