# Supplementary material for: Efficacy of three-dimensionally integrated exercise for scoliosis in patients with adolescent idiopathic scoliosis: study protocol for a randomized controlled trial
Source: Trials. 2018 Sep 10;19:485. doi: 10.1186/s13063-018-2834-x (PMC6131734; doi:10.1186/s13063-018-2834-x)
Supplement: Supplementary file 1 — World Health Organization Trial Registration Data Set. (PDF 152kb) [file 13063_2018_2834_MOESM1_ESM.pdf]

Table 1 World Health Organization Trial Registration Data Set (Version 1.2.1)

Item number	Item	Description	Addressed on page number
1	Primary registry and trial identifying number	Name of primary registry and the unique identifier assigned by the primary registry	3
2	Date of registration in primary registry	Date when the trial was officially registered in the primary registry	3
3	Secondary identifying numbers	Other identifiers, if any Universal Trial Number Identifiers assigned by the sponsor Other trial registration numbers issued by other registries Identifiers issued by funding bodies, collaborative research groups, regulatory authorities, ethics committees/institutional review boards, etcetera.	Not applicable
4	Sources of monetary or material support	Major sources of monetary or material support for the trial (for example, funding agency, foundation, company, and institution)	17
5	Primary sponsor	Person, organization, group, or other legal entity that takes responsibility for initiating and managing a study	17
6	Secondary sponsor(s)	additional persons, organizations, or other legal persons, if any, who have agreed with the primary sponsor to take on responsibilities of sponsorship	Not applicable
7	Contact for public queries	E-mail address, telephone number, and postal address of the contact who will respond to general queries, including information about current recruitment status	2
8	Contact for scientific queries	Name and title, e-mail address, telephone number, postal address, and affiliation of the principal investigator and e-mail address, telephone number, postal address, and affiliation of the contact for scientific queries about the trial (if applicable)	2
9	Public title	Title intended for the lay public in easily understood language 1	1
10	Scientific title	Scientific title of the study as it appears in the protocol submitted for funding and ethical review; include trial acronym, if available	Not applicable
11	Countries of recruitment	Countries of recruitment Countries from which participants will be recruited	5
12	Health condition(s) or problem(s) studied	Primary health condition(s) or problem(s) studied (for example, depression, breast cancer, or medication error)	4
13	Intervention(s)	For each group of the trial, record a brief	

		intervention name plus an intervention description name. For drugs, use the generic name; for other types of interventions, provide a brief descriptive name of the intervention. This name must be sufficiently detailed for it to be possible to distinguish between the groups of a study; for example, interventions involving drugs may include dosage form, dosage, frequency, and duration	5-10
14	Key inclusion and exclusion criteria	Inclusion and exclusion criteria for participant selection, including age and sex	7-8
15	Study type	Method of allocation (randomized/ nonrandomized) and blinding/masking (identify who is blinded) Assignment (for example, single group, parallel, crossover, or factorial) and purpose Phase (if applicable) For randomized trials - method of sequence generation and allocation concealment	5
16	Date of first enrollment	Anticipated or actual date of enrollment of the first participant	16
17	Target sample size	Total number of participants to enroll	13
18	Recruitment status	Pending - participants are not yet being recruited or enrolled at any site Recruiting-participants are currently being recruited and enrolled Suspended - temporary halt in recruitment and enrollment Complete - participants are no longer being recruited or enrolled Other	16
19	Primary outcome(s)	The primary outcome should be the outcome used in sample size calculations or the main outcome used to determine the effects of the intervention For each primary outcome, provide the following: Name of the outcome (do not use abbreviations) Metric or method of measurement used (be as specific as possible) Time point of primary interest	10
20	Key secondary outcome(s)	As for primary outcomes, for each secondary outcome provide the following: Name of the outcome (do not use abbreviations) Metric or method of measurement used (be as specific as possible) Time point of interest	10-12
